# Roserl

**Published:** 1876-10

**Authors:** Marie Orm


*From Treasure Trove.*

### ROSERL.

### An Alpine Scene.

By Marie Orm.

### CHAPTER II.

FOR a moment Roserl stood transfixed. Her anger and resentment gave way for the time, and a nameless horror possessed her. She went to the edge of the precipice, and stooping cautiously over the frightful chasm, peered into the inky depths below. But her eyes could not pierce the dreadful gloom. She called her lover's name, but only the sullen roar of the torrent below answered her; then, becoming enraged again, as well as frightened, she retraced her steps to the hut, and entering, gave way to the mad fit of jealousy that was upon her.

"What care I what has become of him!" exclaimed the frantic girl. "The wretch! the murderer! treat *me* so, and after what had gone before, having betrayed me into confessing my weakness—my love—my mad love for him. But serve me right, goose, idiot that I was, to allow myself to be so overcome as to forget all resolution—to let him see how passionately I was fond of him, ungrateful wretch!" And flinging herself down on the wet threshold of the hut, she lay prostrate on the floor, giving herself up to passionate crying.

The torrent of tears, however, served to calm the impetuosity of her grief. She sobbed on and on, until sobs turned into heavy breathing and heaving, and tears fell abundantly but quietly. Long, long, lay she motionless, and cried—and cried.

In the meantime, the sympathizing weather had reached the pitch of inclemency, and the impetuosity of the storm could not fail to attract her attention in the end. She began to listen to the howling of the wind, the splashing of the rain, peals of thunder which, in their echo from the hills, formed a continued roar. That uproar of the elements seemed in keeping with the feelings of her own tortured breast; it soothed her where nothing else would have brought comfort.

A dreadful thunderbolt close to her, shaking the foundation of the hut, caused her to start from her prostrate position. Her first thought was that the lightning had struck the cowhouse. Erect she stood in an instant, and rushed out to the stables. Nothing seemed the matter there. Her second thought was the precipice, which rocky chaos had several times before been struck and rent by lightning; she could even remember the last time when a new rent had been made in those shaggy cliffs. With a lond shriek she flew to its brink, aud endeavored to look into the depth; a terror-stricken call, which sounded like "Franzl, Franzl," died away in the roar. Surely the girl must have lost her senses, that she did not cry, "Wretch, murderer!"

The streaming rain, however, hid all from view, and blinded her. Clap after clap of thunder, all the horrors of the tempest, were let loose upon her head as she bent down, vainly hoping to discern something in the black hole. "If he does not get out soon, he must be drowned. He can not get out the other way below; it must be flooded; it would be sure death; he knows that." She knew he must come out this way, and what could he do but ask shelter at her hut? Oh, he would do so at anybody's hut, not at her's now—she knew that, too. But if he came near, she would call him in—he would come in, he *must* come in; he could not go a step further in such a storm. Oh, if he but made his appearance! Ha! another clap, so near! The earth shook, a thundering noise followed. An immense rock, not far from Roserl, had been split in two, rent from the ground, and went rolling down the height, disappearing as it dipped into a gray ocean, but heard a long while after as it rolled from one stone terrace to another, until, far down at the bottom, it reached a cleft wide enough to settle in. There the two enormous blocks lie now, one a little above the other, and there they will remain for centuries, tourists admiring their fantastic shape and their picturesque position.

The shock had sent Roserl reeling to the ground. She held her head with her two hands, feeling as if she was to be rent asunder next. But as soon as the first fright was overcome, she managed to lift her head and spring to her feet. Franzl was uppermost in her thoughts; she stood at her door ready to receive him; the next minute must bring him. The path out of the rugged lepth led close by her hut; he could not have passed; he would have had to stalk over her as she was lying across the path.

Half the night passed; the storm had subsided, a calm, healthful rain was noiselessly falling; Nature seemed weepingly to rest from her travail, and Roserl was still watching for Franzl. If he had been hiding in a cave below, he would be coming now. Patter, patter, rain—no other sound.

Roserl felt wearied out and sick at heart; still, no wish for rest or sleep had she.

At last she left her post, and scarcely knowing what she did, she walked across the Alm in the direction of another Alm hut. Nannerl was greatly shocked when she heard the violent knocking at her door. She opened after long hesitation. "Well, Roserl!" she exclaimed, and stood like one petrified at Roserl's pale face. "I feared the guards——"

"Ah! thou hast given refuge from them to a man in thy house. Call him up—call him up at once! I must see him."

Nannerl wanted an explanation, but the impetuosity of Roserl's manner awed her, and she proceeded to the back of the hut, where she put a ladder to the loft, and climbing up, she knocked in a peculiar way upon a board covering the entrance. She had been heard and understood, and retraced her steps. A moment afterward, a long, sleepy figure, fully dressed, yawning and stretching itself, tumbled down the ladder, alighting at Roserl's feet. "Why, Roserl! what on earth can be the matter?"

"Oh, Hansel, come with me at once! Franzl must have met with an accident in the black hole."

"Not he!"

"But I assure thee he went in before the storm began, and has never come out again."

"Franzl is the last man to remain in the loch. He no doubt got out the other way."

"No, no! I know that loch well; there is but one way out of it, and I have watched it all night."

"But he would find another way. He scrambled over the rocks, I warrant thee."

"He could not; it was flooded!" cried Roserl. "The waters came down over the rocks like a waterfall."

"Hm! yes—perhaps so. Well, we can't do anything now. But in the morning I'll fetch the fellows at Feferl's and we'll search the precipice. But thou needst not trouble; be sure, Franzl has spent the night in his own bed."

Roserl heard no more, she was off to Feferl's. She found Feferl dressed, and at her work in the dairy. But although everything indicated her having hidden from the guards some wildschütz, she denied most decidedly having a man in the house, and remained unmoved by Roserl's frantic despair. The fact is, Feferl had her own lover, to whom she was soon to be married, among the party she had taken under her protecting wing; and what were all the Franzls of the earth to her, when her own lover's safety was at stake?

It was daybreak when Roserl returned to her hut, disconsolate, broken-hearted, exhausted. The rain had nearly ceased. Without a moment's pause, she proceeded at once to the black hole. Looking around her, searching in every crevice, she climbed down the steep like a cat. "Franzl!" she called, when she fancied she saw or heard the least thing. But there was scarcely any voice left in her; it had died out with the hope of her heart. If she found him at all, it could but be as a corpse. Rather not see a trace of him, then he might be safe elsewhere. She began to give room to that reviving thought, when suddenly her heart stood still. What could that be lying there, half covered by an overhanging rock?—a human form? "Franzl!" she tried to articulate, but no voice came in reply. In vain she gasped for breath. But now!—could it be—was not that form moving? Could her dimmed eyes be mistaken? Oh, no!

There was an effort to move; one arm was thrown out and then employed to raise the recumbent head, whose features remained hidden from Roserl's sight. "Franzl! Franzl!" her voice broke forth, piercing the air.

"Who is there?" a feeble cry answered from below.

Roserl never knew how she managed it, but in a minute she had got down the terrific height, and was kneeling beside Franzl, looking anxiously into his pale face. Alive he was, but—she could not finish the thought; not a word could she say, but covered that death-stricken face with passionate, repentant kisses.

Franzl only now seemed to recognize her. "My Roserl!" he breathed, faintly.

"Thy Roserl!—thy Roserl! Oh God, have mercy!" cried Roserl. Her head sank down on Franzl's breast, and she wept aloud.

Franzl lifted one arm, but his face was contorted with pain as he did so, and laid it caressingly around the girl's neck. "Oh, don't cry so, Roserl!"

"That is *my* doing," sobbed the poor girl. "I sent thee down the pit!"

"No, no, Roserl; it was an accident. But don't cry so. I am not going to die yet. I have a mind to live a little longer. Only I—I—can't move a bit—horrid pains—something is smashed—only my leg, I believe--and a few ribs—oh!—never mind, Roserl, it will be all right soon, I daresay," and a fainting fit disclaimed all his protestations.

Roserl sobbed despairingly. "Franzl, Franzl! Only tell me thou hast forgiven me—only open thy eyes once more—only look on me once more!" which he, like a good boy, did. He made a frantic effort to shake off the dizziness.

"My own poor Roserl, it was all my own fault. I was mad with rage when I undertook to go down the Dark Hole. Like a blind man, I rushed into sure death, and met with what I deserved. I stumbled like a drunken man; and—before I was aware of slipping—I went down the whole height, with a rapidity that stunned me! The rushing waters woke me up; I should have been drowned there below. I tried to raise myself. I could not at first; but rather than be drowned, I made desperate efforts, and succeeded in dragging myself so far up. Here, with my head under the rock over which the waters fell with a loud rush, I thought I might rest awhile, and then I fainted again. Here I have spent the night, as it seems, Roserl." He paused, exhausted; the deadly pallor again spread over his face, which had been slightly tinged with color whilst he had spoken. Roserl wept on in silence.

He soon opened his eyes again, and a light flush passed over his cheeks. Pointing to the breast-pocket of his torn coat, he said, with a faint look of mischievousness in his eyes, "But I have got the flowers!"

"Oh, give them to me again!" cried the girl; "give them to me. I will never part with them. I will never provoke thee again—*never, never!*"

"And Hansel?"

"I will *never* see him again! I asked him to help me to find thee—and he would not come with me."

Franzl smiled. He seemed quite satisfied, and shut his eyes again allowing Roserl to lavish her caresses upon him. She talked to him in broken, soothing, comforting accents, smoothed his dishevelled hair, kissed his pale forehead a hundred times over and over again. Then she laid her head on his chest, anxiously listening to his breathing, and rejoiced to find his heart beat like a hammer against her cheek—that heart she had feared to find so still.

The break of dawn had passed into daylight, and Roserl was still sitting with Franzl, supporting his head in her arms, resting it on her bosom, watching every breath he drew. He seemed asleep, and Roserl dared not stir. Hansel did not come with the promised help. The sun rose high and higher, his glaring rays penetrated into the depth; it was evident no help was coming. The men must have left the Alm a long time ago, and now be at their work below. What could she do?

It was a great comfort to hold Franzl in her arms, to know that he was reconciled, and now her own in life or death. But what next?

How long could they remain there, perched on that cliff? And she had no idea yet to what extent Franzl might be crippled.

When at last Franzl opened his eyes to notice that it was broad daylight, she informed him that she must leave him for a minute to look out for assistance. Franzl consented—in fact, he was unable to show any resistance.

Roserl untied her apron and wrapped it around Franzl's poor bruised head, the only protection she could give to it before she let it down from her lap. Gently and very reluctantly she did it; but she must put it down again on the hard stone. Oh, how her heart bled!

She ran up to her hut—she hoped against hope to find Hansel there, and the others waiting for her to lead them on in the search. But she found nobody, and she knew if the young men had failed her, there was no help to be found anywhere in the neighborhood. Yet Franzl could not be left in the precipice.

A little while afterward, Roserl was descending the steep, blankets hanging on her arm, and her large, soft feather-bed thrown over her shoulder. When Franzl saw her coming thus equipped, he exclaimed, "Why, Roserl, thou bringest me my bed—I suppose I am to make my home in this hole. And who knows, if I like it, I may winter here."

Roserl smiled faintly; it was no matter of joke with her what she was about to undertake.

"I have come to bundle thee up like a baby, and to carry thee up to my room."

"Carry *me* up!" cried Franzl, starting with astonishment, and then seized by pain, falling back again.

"Dost think I have not the strength? Didn't I carry my big cowcalf out of the Berghloch two years ago, when as yet I had not my full strength? Now I am a grown-up woman—and the other day I carried my father around the garden in fun."

"He surely had not the dead weight of a smashed body like mine. I should weigh upon thee like a stone, not being able to help a bit. I can't move."

"Thou cans't a little, just a little, enough to turn over on to this feather-bed. Look here, could'st thou not do that?" She spread the feather-bed below the rock upon which Franzl was lying. It looked tempting.

"I'll try, at any rate," cried Franzl. With a desperate effort he actually moved around and gave himself a jerk. Down he came upon the bed prepared for him. But the contortions of his face, the bite he gave his under lip, showed what immense pain he suffered in his broken limbs. Poor Franzl! Roserl had no time to express her compassion. She was desperate, too.

She set to work with energy, first composing his limbs straight on the couch, then wrapping the bed around him, as they do with babies in this country, turning blankets round and round over the feather-bed, and lastly tying the long towels over the firm bundle, until Franzl looked like a white prostrate column.

Franzl offered not the slightest resistance. The truth is, he had fainted away during the operation.

Roserl scarcely knew whether it was a living man or a corpse she was bundling about.

When he was ready, and could not have moved a limb even if he had shown any inclination to do so, Roserl rested awhile to rally all her strength. Then she drew a long breath, and proceeded to load Franzl on her back. Poor Franzl! his head hung over her shoulder; his feet, well preserved by the wrappings, dragged on the ground. However, he was on his progress out of the pit.

Roserl by supernatural strength had managed to reach the footpath, and now slowly, slowly, bent in two, walking with her hands as well as her feet, she proceeded upward. When her strength failed, she remained for a minute lying flat under her load, with her face in the dust, then, with renewed vigor, began to creep again. So, crawling, creeping, dragging, she brought her burden higher and higher up. But it was a wearisome road; and many and fervent were the short prayers her sore heart sent up to Heaven during that toiling progress.

The longest lane comes to an end, they were nearing the top. Courage, Roserl! One more rest, another effort, and she was out of the precipice. Only a few more steps; of course a long time was required to drag over those few steps, but at last they were at the door. Another gasp for breath, they were safely in, and now for the last and final effort. Roserl rose with her burden from the ground, she lifted it up, and laid it gently down upon her bed. Then all consciousness left her, she sank down on the floor at the foot of the bed in a deep swoon.*

* The above circumstance is a fact

So the boy found them who happened to come up soon after, that afternoon, in order to cart the provisions of milk, butter, and cheese down into the village. At first he thought them both dead and gone, and expressed his feelings by loud shrieks of distress. But soon he saw the bundle moving a little as if breath was still left in it, and being a sharp, courageous little chap, he investigated the matter before making up his mind to run away. The truth that these two people might be in a faint only—for there was no blood to be seen indicating murder—struck him forcibly; and the consequence was that Franzl and Roserl had the contents of a large water-tub emptied upon their heads, which judicious proceeding of the boy's was not without the anticipated effect. It revived them both, but Roserl alone was soon upon her feet, while Franzl only expressed by groanings that he was somewhat tired of his narrow prison. When Roserl, although staggering and shaking all over, untied Franzl's wrappings and tried to make him comfortable on the bed, she could not be mistaken about the nature of his injuries. His leg was broken, and no doubt some of the ribs, besides other damages she might not know of.

The boy was instantly dispatched to a neighboring valley for the wise shepherd, whom people consulted in preference to the old village surgeon. Michel, the shepherd, was famous for his wonderful skill in curing breakages of any kind—be it man's or beast's bones, legs of tables and chairs, earthen pots or china-ware.

It was midnight before he came, and Franzl was in a high state of fever. However, Michel was in no way disconcerted. With the dexterity of an experienced man, he bandaged the broken creature up—not without first having applied his wonderful balm.

The whole performance had taken very little time; and after administering a draught to his patient, he departed, recommending Roserl to get a good sleep, and promising to come again next evening. The good doctor had to attend to his sheep before daybreak.

He had scarcely left when Franzl fell into a sound sleep; and Roserl, sitting on blankets on the floor, leaning her head against the pillow on which Franzl's was resting, slept soundly too.

The boy had attended to her wants. He had made a large fire on the hearth, had dried all the wet clothes, cooked a supper for Roserl and himself, and attended to the cows as best he could. Then, in the morning, without having taken any rest, he made up the fire again and left Roserl's broth ready for her on the hearth; then, with another look to see whether they were warm and comfortable, he commended the sleepers to God's Fatherly care in a short prayer, and left the Alm to carry down the provisions and the astounding tale. No doubt to-morrow would see Roserl's mother, aunts, cousins, and godmother on the Alm; but as yet the lovers were alone. The shepherd did not fail them next evening, and found Franzl's state as favorable as circumstances would allow. His view of the case greatly comforted Roserl.

The next night, when Franzl, opening his eyes after some hours' refreshing sleep, met Roserl's gaze watching him, he could smile cheerfully. The ghastly look had left his face, there was even a sparkle of his old good-humor in his eyes, as he said:

"I have brought the Edelweis out of the hole, though!"

"No," said Roserl, mischievously, "thou hast not! *I* brought it out, and thee with it; and now I have an undisputable right to possess you both." And then she kissed his lips to prevent him from answering. The shepherd had strictly forbidden talk.

Our tale is at an end. Franzl's broken bones healed as well and as quickly as his strong and thoroughly healthy nature permitted; the pure air of the Alps is an invigorating physic—and another was Roserl's devoted care. Roserl's mother and other female relatives offered assistance, which was thankfully accepted to a certain extent, but Roserl would not give up her post as head nurse to anyone.

Franzl was as merry as a lark in his bed, tied down as his limbs were. He was kept quiet by Roserl's sweet persuasion and her fond caresses; the doctor had strictly forbidden excitement of any kind. The task was not easy, however; when all other means failed, Roserl would put her pretty, soft arms around his neck, and force the naughtiest, wickedest of boys to rest his dark, curly head on her bosom, and fondly stroking it she would sing a lullaby—such as they soothe the babies with. Franzl might struggle a little, but Roserl was now stronger than he—and so he laughed, obeyed, and was happy. Some time passed before he could be removed to his father's hut. It was then time to drive the cattle down, and they all went together.

By Christmas all was right again, everybody having recovered health and strength. There will be no more sommerfrische on the Alm for Roserl; for at Easter there will be a wedding.

THE END.

